# Organizational issues for the lean success in China: exploring a change strategy for lean success

**DOI:** 10.1186/s12913-019-3907-6

**Published:** 2019-01-24

**Authors:** Tian Gao, Bruce Gurd

**Affiliations:** 1 Planning and Finance Department, Jinan Central Hospital Affiliated with Shandong University, Jinan, 250013 China; 2 University of South Australia, Adelaide, Australia; 3 Australian Centre for Asian Business, University of South Australia, Adelaide, Australia

**Keywords:** Lean application, Healthcare, Chinese hospital, Systematic review

## Abstract

**Background:**

The purpose of this paper is to explore a change strategy for lean success in hospitals based on a comprehensive review of the Chinese literature.

**Methods:**

The methodology is a systematic review of the Chinese literature which identified 212 case study papers about lean implementation. We did a thematic content analysis of the 212 papers.

**Results:**

Lean applications in Chinese hospitals show significant increases and are mainly used in the fields of outpatient services, operating rooms, pharmacy and logistics. Most hospitals applied lean as a single project but some were beginning to use lean as a systemic path for improvement with an emphasis on lean and strategy. The main goals were to increase the operating efficiency and reduce operating costs. Patients were not central to lean applications. Chinese hospitals appear to lack a full understanding of lean. Four factors appear to be critical for lean success - organizational leadership, adequate technology, stakeholder involvement and individual and organizational benefits. The relationship of these factors changes over time.

**Conclusions:**

This is the first paper to provide a comprehensive view of lean application in Chinese hospitals. The findings presented in this paper provide a systemic evidence to the application of lean in healthcare.

## Background

Lean, also known as lean thinking, and the Toyota Production System, is a process reengineering philosophy developed to successful practice by Toyota and consisting of a set of strategic guiding principles and operational tools [1–3].

An important concept in lean is to divide activities into added value and non-value-added value. Value-added activities are considered activities that satisfy a customer’s demand for a product or service; all other activities are non-value-added. By using lean tools, such as value stream maps, organizations can identify activities and bottlenecks that have no added value and provide standardized solutions to these problems. Lean management can create an organization that can accomplish more and better tasks with less time, less human support, less cost, less space, less trauma and fewer mistakes [4]. Originating in manufacturing industry; hospitals have become important sites for lean applications as the result of the need for performance improvement. Lean is considered a solution to meet the efficiency and productivity needs of improving the quality of health services [3]. From the initial application of lean thinking in hospitals to the present, there have been more than 10 years of history [5], and it has been adopted rapidly in health care settings with the goal of supporting the development of a better, value-based organization [6–8]. Many studies claim that applying lean has achieved good results in improving hospital performance [9, 10]. The barriers to lean implementation have led to a slower adoption of lean in hospitals [6]. Organizational issues surrounding the successful implementation of innovation are critical, but there has been insufficient research attention [2].

Since the middle 1990’s, as China’s medical reform has deepened, hospitals have been facing increased pressure to improve medical quality and operational efficiency. In this context, some Chinese hospitals have used lean to improve their performance.

This paper explores organizational issues for the lean success in healthcare through a review of the application of lean in Chinese hospitals based on a systematic survey of the existing literature. The next section provides a review of the literature on lean, introduces the Cresswell and Sheikh model [11] of organizational factors, and introduces the context of Chinese hospitals.

### Lean

Prior studies show the positive outcomes of the application of lean in healthcare, such as improving quality of care, safety, increasing patient and staff satisfaction, achieving productivity and cost efficiency, and better financial outcomes [12, 13]. Some problems in lean practices have been identified. Inconsistent with the origins of Lean in Japan, it has not employed as a systematic approach, a philosophy, and mainly used as a technical fix of practices [14] without a supportive culture for facilitating the lean implementation [15].

While there is a significant literature, systematic evidence of the application of lean is limited [16–19], and further research is needed. There is very limited research done on the application of lean in healthcare in China [16, 20, 21].

### Organizational issues for successful lean implementation

Compared with other industries, the healthcare industry is much more complex, lean concepts have not penetrated all its aspects in hospitals [22]. Many studies have explored factors for the successful implementation of lean, such as the support of each level of management [23], organizational leadership [24], technical support from external agencies [25], internal consultants’ support [16], creating cultural change [24], fostering a long-term view of continuous improvement [14, 22], emphasizing the benefits of the project to the organization, such as patient safety, patient satisfaction, and the hospital’s business and economic performance [14]. Organizational readiness is considered a prerequisite for the initiation and subsequent implementation of lean projects [12]. Successful implementation of lean requires attention to factors including a strong leadership team for lean support, training and stakeholder involvement [22, 25], the basic stability of the organization [26], connecting lean with strategy and establishing a measurement and reward system linked to lean goals [22].

**Fig. 1:**
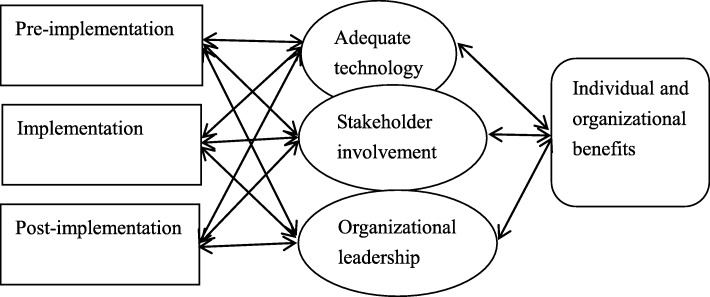
The interaction of technological, social and organizational factors in hospital information technology innovation over time (adapted from Cresswell, K. and A. Sheikh, Organizational issues in the implementation and adoption of health information technology innovations: An interpretative review. International Journal of Medical Informatics. 2013; 82(5): 73–86)

Figure 1 presents a model adapted from Cresswell and Sheikh [11] which connects the factors that affect the successful implementation of a technology innovation including social factors, technical factors and organizational factors; we have extended this to a managerial innovation such as lean. We extend the idea of “adequate technology” to the spread of lean tools available to the hospital. Not only are these factors related but the relationship changes at different points of the implementation. The exact relationship between these dimensions is not clear and further work is needed in this field [11, 27].

### The Chinese hospital context

With the expanding coverage of China’s medical insurance over the last ten years, China’s hospitals are facing increasing pressure because of limited growth in medical resources but, at the same times, a growth in patient outcomes. From 2005 to 2015, the number of outpatients and inpatients in China have increased 121 and 193% respectively [28] whereas resources have increased at a much lower pace with the hospital buildings only increasing by 75%, beds by 108%, and staff by 65%.

During this growth the Chinese government has been continuing to attempt reform to ensure that hospitals provide better services at reasonable prices including the 2005 Ministry of Health ‘Administration Year’ [29]. In 2009, the Chinese cabinet passed a new health care reform plan which increased competition with a reduction of profit from pharmaceuticals, increased patient choice and growth of medical insurance to at least 90% of the entire Chinese population by 2011 [30, 31]. Medical insurance has become the major source of hospital revenues in China [32].

The use of payment methods of medical insurance payments, such as fee-for-services and total budgets has forced the hospitals to reduce their costs and improve their productivity. According to the hospital reform policy [33], before the end of 2015 Medical insurance payment reform will cover all public hospitals, covering more than 30% of the hospital discharges of public hospitals at a county level. By 2017, China will fully implement a compound payment method of which DRGs (diagnosis-related disease groups) will be the main method, capitation and per diem payments will be supplementary. The medical insurance department will be paid according to the type of disease. For a disease type, no matter what kind of medical items happen, the one-time payment will be made according to the price of the disease type. If the medical cost exceeds the payment price of the disease, the medical institution will have to bear the cost of exceeding the payment price.

These all point to the need for Chinese hospitals to effectively control unreasonable cost increases, strengthen medical quality management, further improve medical efficiency and decrease medical costs.

There has been a very important policy change since the health care reform in the mid-1980s, in that the Chinese Government tightened the fiscal budgets for public hospitals and other health care institutions [34, 35]. The government only finances part of the operating outlay of public hospitals and the subsidy from the government for public hospitals was cut to 14–30% of total hospital spending, which is only basic personnel salaries and new capital investments [36, 37]. The public hospitals were given more economic autonomy to find new financial resources and generate more capital and profits from other services and pharmaceuticals to cover the gap between their total spending and the government’s financial subsidy [31, 38, 39]. In this environment innovations in health delivery are likely, and lean management is attractive for this focus on improving operations performance as well as drivers of cost effectiveness.

The first research question is, what is the current state of the lean implementation in Chinese hospitals?The focus is on the trend, approaches, outcomes and problems of the application of Lean in Chinese hospitals. Furthermore, for exploring the successful lean implementation, our second research question is, what are the relations between technological, social and organizational dimensions at different phases of implementation?

## Methods

This study used quantitative methods including print media indicators (PMI) and content analysis. We analyzed PMI following Braam et al.’s [40] approach in the study of Netherlands. The resources surveyed were Google Scholar, Baidu scholar, and three Chinese academic data bases – Wanfang Data, CQVIPVIP (Chongqing VIP Information Co., Ltd.) database and CNKI (Chinese National Knowledge Infrastructure) database to find published refereed journal papers using key words related to lean in Chinese hospitals before 2017. 379 journal papers were found, with 212 case studies of implementations and 167 theoretical papers arguing for the virtues of lean and exploring issues in its use. Around 127 hospitals had claimed they had implemented lean and introduced their experiences. 20 hospitals have published more than one article which provides relatively more comprehensive data on the application of lean.

Through the use of thematic content analysis, we refined the data from the 212 papers of case studies of lean implementations around the trend, approaches, the understanding of lean, lean tools used in practice, outcomes and problems. Then we coded, classified and the data in an Excel spreadsheet for further analysis.

## Results

### Social aspects

#### The trend of papers published on lean

**Fig. 2:**
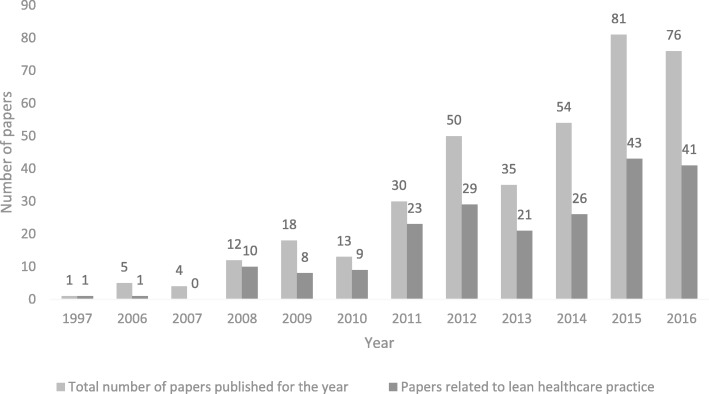
Growth trend of published papers on lean in Chinese hospitals

Figure 2 indicates the growth of the published papers on lean in Chinese hospitals. After the first paper reported the lean practice in Chinese hospitals was published in 1997, the second paper appeared eight years later. In 2008, papers were published at a more rapid rate than in 2007. The year 2009 can be seen as a milestone of the diffusion of lean in Chinese hospitals. In 2011, there was a jump in both the total number of papers and the number of papers related to the lean practice. In 2012, 2014, and 2015 the trend kept rising. The data represents a rapid rise of the diffusion and implementation of lean in Chinese hospitals.

#### The patterns of lean implementation

**Fig. 3:**
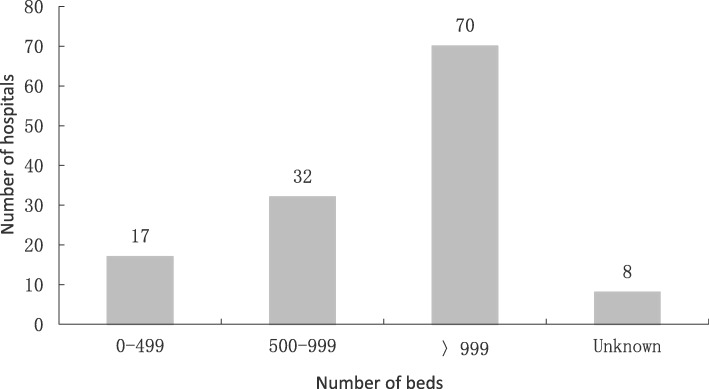
Hospital size

Most of the 127 hospitals that have used lean have more than 500 beds (see Fig. 3), the biggest one has 4398 beds. Most of them (125) are public hospitals. Of the 127 hospitals, 6 hospitals gained external consultants’ help to implement lean. They published 48 articles of lean application which occupied 23% of the total lean practice articles.

**Fig. 4:**
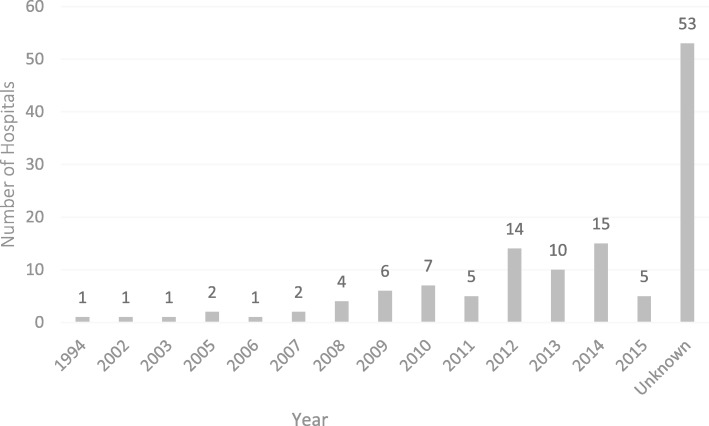
The starting year of the use of lean

Fifty one provided the information at the time they started to use lean (see Fig. 4). The first reported user of lean in Chinese hospitals is a workers hospital of the biggest automobile manufacturing group in China, so it is not surprising that they learned to use the Toyota production system in 1994 [41]. Most hospitals started to use lean between 2008 and 2015. To summarize, excluding 2015, at least 69 hospitals have had more than two years of experiences using lean.

### Technical issues

Most of the hospital (115 hospitals) recognized lean as process optimization tools which can eliminate waste. Eighty five looked at lean as continuous improvement and a very small group saw it as a strategy and a philosophy. Some papers only used the word “lean” in the title, but showed no particular understanding of lean [42]; it seems that lean is anything. Much traditional work, putting on the coat of lean, has become a lean improvement. Hence, what lean is needs to be clarified in Chinese hospitals.

#### Hospital fields in which lean has been implemented

Only seven hospitals claimed to have applied lean across the whole hospital [41, 43–47] but not always covering all staff. In one case a hospital had implemented lean for 3 years, but it had not spread to all staff [45].

Specifically, lean was most widely used in pharmacy and operation room (28 hospitals) [48, 49], and then outpatient process optimization (22 hospitals) [50, etc.], outpatient service and nursing management (14 hospitals respectively) [51]. It was also used in the field of integrated management, for example, financial management [52], human resource management [53] and hospital culture Change [54] (see Table 1). Compared with hospitals in Western countries, there are two distinct differences. The first is that pharmacy plays an important role in hospital operations and it also an important part of the services for the patients. Second, China’s hospitals usually have a large number of outpatients. For example, Changhai Hospital of Second Military Medical University has around 11,000 outpatient visits per day [50], and in Guangdong Province the Traditional Chinese Medical Hospital the number of outpatient pharmacy prescriptions is 8000–12,000 per day in 2009 [55]. So it is not surprising that the procedure optimization related to pharmacy and outpatient services are the most areas using lean in Chinese hospitals.

**Table 1:** Hospital fields in which lean has been implemented

Hospital units	№	Supportive management	№
Pharmacy	28	Nursing management	14
Operating Room	22	Logistic material supply	12
Outpatient service	14	Specialty not specified	8
Clinical Laboratory	6	File management	6
Specialty not specified	6	Equipment management	6
Health examination	5	Financial management	3
Emergency Department	5	Human resource management	2
Ward management	4	Medical service quality	1
Radiology Department	4	Medical insurance management	1
ICU (Intensive Care Unit)	2	Propaganda work	1
Department of Obsterics	2	Medical safety management	1
Department of Respiratory	2	Identity management	1
Digestive endoscopy center	1	Hospital culture	1
Pediatrics Department	1	health care for cadres	1
Pre hospital emergency management	1	Medical Waste Management	1
Anesthesia recovery room	1		
Department of general surgery	1		
Blood Transfusion Department	1		
Dental Department	1		
Cardiac surgery	1		
Department of Orthogedics	1		
Department of Radiotherapy	1		
Department of Gynaecology	1		
Department of Neurology	1		
Department of Neurosurgery	1		

#### The main lean tools used in Chinese hospitals

In the lean practice of 127 hospitals, 39 lean tools are mentioned (See Table 2). Among the 39 tools, 17 were used by only one hospital. 5S (sort, set in order, shine, standardize and sustain) (and related 6 s (sort, set in order, shine, standardize sustain and safety) and 8 s (sort, set in order, shine, standardize, sustain,safety,save and study)) is the most widely used tool (31 hospitals), then Value Stream Map (24 hospitals) and Fishbone Diagram (24 hospitals) (See Table 2).

**Table 2:** The frequency of methods or tools of lean used in Chinese hospitals

Rank	Methods or Tools	№	Rank	Methods or Tools	№
1st	5S (Sort, Set in order, Shine, Standardize, Sustain) related	31	17th	Kanban	2
2nd	Value Stream Map	24	17th	Pareto analysis	2
2nd	Fishbone Diagram	24	23th	Just In Time	1
4th	Six Sigma	19	23th	Push	1
5th	Work-out	16	23th	Transaction improvement	1
6th	Visual management	12	23th	Zero inventory	1
7th	DMAIC (Define, Measure, Analyze, Improve, Control)	11	23th	Jidoka	1
8th	Brainstorming	10	23th	Water spider	1
8th	PDCA (Plan, Do, Check, Act)	10	23th	Takt Time and One piece flow	1
10th	Spaghetti Chart	8	23th	Scenario simulation	1
10th	QCC (Quality Control Circles)	8	23th	Monte Carlo simulation	1
12th	SOP (Standard Operation Procedure)	7	23th	SIPOC (Supplier, Input, Process, Output, Customer)	1
13th	Pull System	5	23th	Three-point estimation	1
14th	ECRS (Seliminate, combine, rearrange, simplfify)	3	23th	RPN (Risk Priority Number)	1
14th	Standard Work	3	23th	CTQ (Critical-To-Quality)	1
14th	Flow Chart	3	23th	Control influence matrix	1
17th	Location management	2	23th	Failure Mode and Effect Analysis	1
17th	5WlH (Who, When, Where, What, Why, How)	2	23th	GRPI (Goal, Rote, Process, Interpersonal relationship)	1
17th	Pokayake	2	23th	Root cause analysis	1
17th	80/20 principle	2			

The top three are the tools for site management, waste elimination and quality improvement. The two hospitals with the most lean tools, using 15 and 13 tools respectively, are Taihe Hospital and Nanfang Hospital which are supported by external consultants. This indicates that the lean management knowledge and technology in the Chinese hospitals needs to be popularized. The most frequently used top ten lean tools are process improvement and the improvement of the physical area. This is consistent with the characteristics of the Chinese hospitals in 4.3.

### Organizational factors

#### The approaches to the application of lean

In addition to the eight hospitals which applied lean across the whole hospital^1^ the other 119 hospitals only used lean in some units. Most of them implemented lean as a project. Around 80% of hospitals chose to do pilots first, and then gradually promoted lean practice across the whole hospital.

It is argued that the selection of lean projects mainly depends on whether the project itself can meet the relevant characteristics and conditions: first, the cycle of the project is short and can yield faster results; second, less resource will be involved in the project, its process is relatively independent; third, the project can have a large impact and play a benchmark role; fourth, the project manager is passionate, staff agree with the project [44].

These points reflect the difficulties in the implementation of lean. That is, lean implementation is a long-term process, needs a certain time to display the effect, often needs to cross the department to implement, needs to master certain knowledge, requires to get everybody involved and needs a supportive culture.

#### The outcomes of the application of lean

**Fig. 5:**
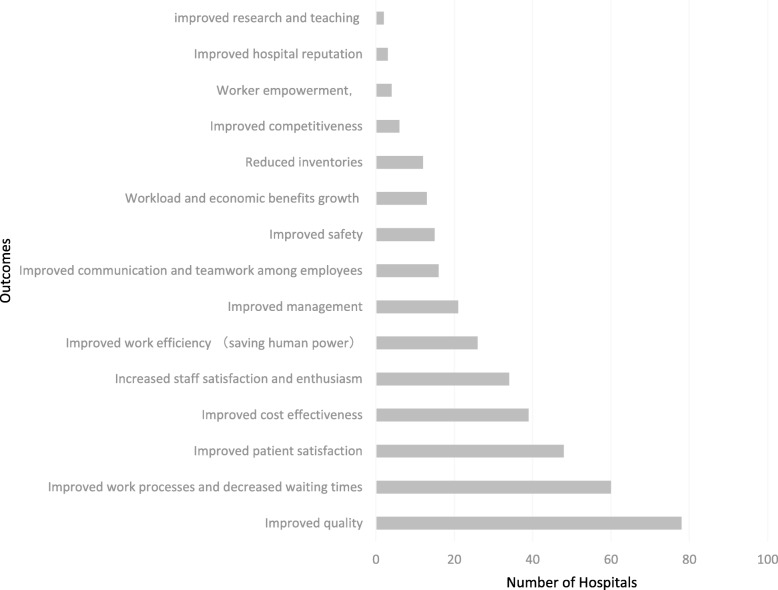
The outcomes of the lean implementation in Chine hospitals

In this study, nearly all of the hospitals provided information regarding the outcomes of the application of Lean (see Fig. 5). All the outcomes are positive; unsurprisingly, failures are not reported. Reported outcomes of the 127 hospitals are supported by specific data.

Among the 124 hospitals that provided information the outcomes are shown in Fig. 5, 78 improved medical quality control; 60 claimed positive results in the indicators relating to improved work processes and decreased waiting times; 48 hospitals improved patient satisfaction; 39 hospitals improved cost effectiveness and 34 hospitals claimed that they have improved staff satisfaction and enthusiasm; 26 hospitals have improved work efficiency; 21 hospitals have achieved an improvement in management and 16 claimed that communication and teamwork among employees have improved; 15 hospitals mentioned improved safety. It is worth noting that there are 13 hospitals claiming that they have achieved an increase in workload and economic benefits, and 12 hospitals have reduced their inventories. As Chinese public hospitals rely mainly on their own income they are largely driven by economic interest.

Less commonly they reported improved competitiveness, worker empowerment, improved hospital reputation and improved research and teaching.

These findings show that the main focus of the hospitals on the application of lean is to optimize processes to improve operational efficiency and not to meet the needs of patients. For example, “As the hospital business continues to rise, for example, the hospital outpatients increased from 665,000 person-time in 2006 to 1,052,000 person-time in 2009. However, over the same period, the number of hospital staff only increased from 1020 to 1126….we realized that we must improve our work efficiency and reduce the occurrence of errors” [45]. The goal of another hospital to apply lean is to “reduce operating costs, improve efficiency” [53]. Similarly, the purpose of a hospital in optimizing operating theater procedure is to reduce the waste of human resources and material resources, improve work efficiency and staff satisfaction degree [56].

#### Inter-relations of technical, social and organizational dimensions in the lean implementation process

Twenty six hospitals mentioned the importance of training in lean implementation Twenty two hospitals believed that cultural support is an important factor in the successful implementation of lean. This might include staff involvement as noted by 7 hospitals and support and empowerment of the top leaders noted by 6. Three hospitals referred to the need to combine incentives and lean implementation. Cross sector cooperation was also mentioned.

Twenty two articles mentioned the relationship between lean and culture. Lean management is considered as an idea, an attitude, and a reflection of culture [53]. The hospital lean culture is considered as an important link in the hospital lean management chain [57]. The deepening of health care reform and the intensifying competition in the medical service market put forward higher requirements to the hospital management, therefore, “*hospital management will need to learn some advanced business philosophy and technology to improve the level of management*” [58], lean management has become the choice of some hospital administrators [58]. However, because of the application of new management concepts and methods, it is necessary to change the existing habits, inevitably bringing about conflicts with the original culture. Therefore, Christopher, et al. [59] pointed out that before the lean technology is widely applied, there are many cultural barriers that must be overcome, including transferring a manufacturing concept into the medical sector. Geng et al. [58] argues that the cultural change brought about by the implementation of lean is not a cultural alternative to another culture, but a fusion of innovation based on the original culture. They emphasized that the main reason for the hospital to choose lean is that its inherent characteristics of hospital culture accord with the lean management concept [58].

## Discussion

Public hospitals have had to pay more attention to justify the use of limited resources and improve operational efficiency; these appear to have resulted in the demand for Lean. The evidence for this is that the most important outcomes are the increase in workload or economic benefits.

Western literature suggests that consultants, due to the lack of medical background, are not easily accepted by medical personnel in lean implementations [60]. In China, hospitals tend to use consultants for lean which can suggest greater acceptance as well as promotion of their services. This differs from previous studies that showed that consultants did not play an important role in the diffusion of the balanced scorecard in Chinese hospitals [61]. This may because the technical methods of lean are from manufacturing industry which are not easily understood by medical professionals.

Most of the 74 hospitals have more than 500 beds. Previous studies have indicated that larger organizations are more likely to use specialized and sophisticated management control systems [62]. As larger organizations have more decentralized organizational structures and more specialized tasks and complicated processes, more advanced management control systems are required by these larger organizations [62]. Larger hospitals have more patients, so there is more demand for optimizing the process. They also have more resources to invest in processes such as lean. But as the knowledge of lean grows, more small hospitals have begun to use lean to improve their management.

However, in healthcare, the lack of a standard definition of the customer value of lean [15, 63] will impact the outcomes of lean applications. The findings presented in this paper suggest that lean in Chinese hospitals is mainly used to eliminate process waste and improve the efficiency of the service for patients, which seem to be inconsistent with the patient as the centre of the lean concept. But there was no evidence of using it for over-treatment, the great waste of medical resources. This is mainly due to the heavy financial pressure faced by Chinese hospitals. In this study, 26 hospitals claimed that they have achieved improved cost effectiveness, but there are no hospitals who claim that they have successfully reduced the expenditures of patient care. Patient satisfaction increased mainly because of reduced waiting time. One of the reasons is that health expenditure control is also a difficult point for hospitals around the world, and another reason is that if the hospital reduces the expenditures of patient care, the hospital’s revenue would be possibly reduced as well, so they prefer to reduce operational cost to maximize their operational benefits. Hence, it is not difficult to understand that patients are not the real focus in the application of lean in most Chinese hospitals.

In comparison with Western hospitals, Chinese hospitals focus more on fields with a high flow pressure such as outpatient, pharmacy, and other related units. All the Chinese hospitals have their own pharmacy, and have more outpatient visits than western hospitals.

Balle and Regnier [64] argue that lean should be considered as a complex thinking system; a philosophy, not just a combination of tools. Some authors thought that applying lean as a project will have a negative impact on the achievement of continuous improvement [15, 65]. We found only 6 hospitals have used a systemic way to implement lean in their hospitals, most hospitals applied lean as a project. But the results of this study demonstrate that the application of the project can be perceived to achieve continuous improvement. For example, 35 hospitals believe that the implementation of lean projects can achieve continuous improvement.

Theoretically, a systematic application of lean can achieve better results. But in healthcare, medical personnel’s professional culture requires them to see the evidence [66] because doctors who have a strong tradition in evidence-based care are willing to accept the change based on good evidence [66]. Therefore, the single project implementation has a positive significance as it can provide a quick effect and promote the application in the whole system. Although its effect is limited compared with the systematic application, it can effectively promote lean in healthcare, and achieve sustainable results. Therefore, partial application is not a limitation. It is an inevitable path for the development of lean in the healthcare industry. Even a hospital that has systematically applied lean for 3 years has not fully implemented lean into every sector [45], which illustrates the implementation of lean is a long-term work. As Radnor et al. noted that the evolution of lean in healthcare will be similar as in manufacturing industry, “from shop-floor based tools, to a process view, and ultimately, to a holistic understanding of pathways across organisations if the benefits of Lean are to be fully realised” [15].

Although the study found that 39 lean tools had been used, most of the tools were mastered by a very small number of hospitals. Lack of lean knowledge is still an important issue in Chinese hospitals, which will directly affect its promotion. Due to the holistic and long-term nature of lean implementation, and the lack of knowledge and technology, the emergence of fake lean [67, 68] is not surprising.

With the enhancement of national health care reform, the implementation of public hospital reform and the promotion of market competition, Chinese hospital is faced with the urgent need to improve the management level quickly, but they lack the scientific method to solve the specific management problems, so the application of foreign advanced management tool such as lean has become a trend.

In addition, because of the impact of professional culture, medical staff are willing to accept a new method after they have seen the evidence of success. Therefore, it is not surprising that lean applications in Chinese hospitals are mostly localized rather than idealized whole hospital applications.

For a long time, due to the lack of the pressure of market competition, Chinese hospitals, especially public hospitals, rely on vague experience based management based on self-discipline culture. When this kind of fuzzy experience management based on self-discipline culture needs to be changed to scientific and specific lean management through standardization, the change of people’s concept is very important. The change of concept requires a long process, which is difficult to be achieved through the implementation of a single lean project. In the future, it will be an inevitable trend to realize the integration of the external lean theory and traditional Chinese culture through the systematic and strategic lean implementation so as to achieve sustainable improvement in line with the lean methodlogy.

## Conclusions

The findings in this paper show that the broader context has an important influence on the application and the application of lean in Chinese hospitals. The application of lean in Chinese hospitals showed a rapid upward trend, which was mainly used in the fields of outpatient services, operating room, pharmacy, logistics and so on. The main body of the application of lean is in large hospitals with more than 500 beds. With the further popularization of lean, more and more small hospitals have joined lean applications.

Most hospitals applied lean as a project. The trajectory is that hospitals are using lean as a systemic path and beginning to emphasize the relevance of lean and strategy. They still lack sufficient knowledge, which is the factor that affects the promotion of lean in Chinese hospitals. Consultants’ participation played an important role in the popularization of lean tools and knowledge. Although lean management emphasizes the customer as the center to achieve improvement, the main effect indicates that lean was mainly used to improve the operating efficiency and reduce operating costs in Chinese hospitals. Patient spending and patient safety were not paid enough attention which is related to the operating pressures of Chinese hospitals.

In reviewing the publications, this paper firstly provides a general picture of the lean in Chinese Hospitals. The reflections on the present level of practice of the lean are useful for both academics and practitioners. The outcome of this study will be of significance for the practices of the BSC in hospitals.

In this study, we used a literature search to find out which hospitals had applied lean. This method has limitations, as implementations viewed as unsuccessful are not likely to be written up and published. And the analysis was limited by using information from papers which sometimes did not go into great detail, although we chose to do a small sample size on-site interviews. In the future, based on these findings in this study, there is an opportunity to conduct a survey of a larger scale to include all Provinces in China to get a better understanding on the lean application issues in Chinese hospitals.

## Abbreviations

CQVIP: Chongqing VIP Information Co., Ltd
CNKI: Chinese National Knowledge Infrastructure
CTQ: Critical-To-Quality
DMAIC: Define, measure, analyze, improve, control
DRGs: Diagnosis related disease groups
ECRS: Seliminate, combine, rearrange, simplfify
GE: General Electric Company
GRPI: Goal, rote, process, interpersonal relationship
ICU: Intensive care unit
PDCA: Plan, do, check, act
QCC: Quality control cycle
5S: Sort, set in order, shine, standardize, sustain
5WlH: Who, When, Where, What, Why, How
6S: Sort, set in order, shine, standardize sustain, safety
8S: Sort, set in order, shine, standardize sustain, safety, save, study
SIPOC: Supplier, Input, Process, Output, Customer
SOP: Standard operation procedure
RPN: Risk priority number

## Footnotes

1 One hospital did not provide specific information on the lean application.
